# Machine intelligence in healthcare—perspectives on trustworthiness, explainability, usability, and transparency

**DOI:** 10.1038/s41746-020-0254-2

**Published:** 2020-03-26

**Authors:** Christine M. Cutillo, Karlie R. Sharma, Luca Foschini, Shinjini Kundu, Maxine Mackintosh, Kenneth D. Mandl, MI in Healthcare Workshop Working Group, Tyler Beck, Elaine Collier, Christine Colvis, Kenneth Gersing, Valery Gordon, Roxanne Jensen, Behrouz Shabestari, Noel Southall

**Affiliations:** 1 National Center for Advancing Translational Sciences, National Institutes of Health, Bethesda, MD USA; 2 Evidation Health Inc., San Mateo, CA USA; 3 Department of Radiology, The Johns Hopkins Hospital, Baltimore, MD USA; 4 University College London, London, UK; 5 Alan Turing Institute, London, UK; 6 Computational Health Informatics Program, Boston Children’s Hospital, Boston, MA USA; 7 Departments of Pediatrics and Biomedical Informatics, Harvard Medical School, Boston, MA USA; 8 National Cancer Institute, National Institutes of Health, Bethesda, MD USA; 9 National Institute of Biomedical Imaging and Bioengineering, National Institutes of Health, Bethesda, MD USA

**Keywords:** Therapeutics, Diagnosis, Medical imaging, Disease prevention, Public health

## Abstract

Machine Intelligence (MI) is rapidly becoming an important approach across biomedical discovery, clinical research, medical diagnostics/devices, and precision medicine. Such tools can uncover new possibilities for researchers, physicians, and patients, allowing them to make more informed decisions and achieve better outcomes. When deployed in healthcare settings, these approaches have the potential to enhance efficiency and effectiveness of the health research and care ecosystem, and ultimately improve quality of patient care. In response to the increased use of MI in healthcare, and issues associated when applying such approaches to clinical care settings, the National Institutes of Health (NIH) and National Center for Advancing Translational Sciences (NCATS) co-hosted a Machine Intelligence in Healthcare workshop with the National Cancer Institute (NCI) and the National Institute of Biomedical Imaging and Bioengineering (NIBIB) on 12 July 2019. Speakers and attendees included researchers, clinicians and patients/ patient advocates, with representation from industry, academia, and federal agencies. A number of issues were addressed, including: data quality and quantity; access and use of electronic health records (EHRs); transparency and explainability of the system in contrast to the entire clinical workflow; and the impact of bias on system outputs, among other topics. This whitepaper reports on key issues associated with MI specific to applications in the healthcare field, identifies areas of improvement for MI systems in the context of healthcare, and proposes avenues and solutions for these issues, with the aim of surfacing key areas that, if appropriately addressed, could accelerate progress in the field effectively, transparently, and ethically.

## Introduction

Machine Intelligence (MI) has increased in use and importance in many industries, including agriculture, transportation, finance, military, and the criminal justice system. In healthcare, the potential of MI to improve patient outcomes—across the spectrum from clinical research through hospital care—is significant^1^, but its development and implementation remain in the early stages of maturity. A number of unique challenges have impeded progress, from healthcare-specific data sharing barriers to the development, adoption, and implementation of MI systems in clinical care workflows in an effective, transparent, and ethical manner^2^. Addressing these barriers at this point in time, prior to wide-scale implementation, will allow research to take advantage of lessons learned from other sectors and realize the full promise of MI in healthcare settings, while avoiding the pitfalls, easily intensified due to the inherently intimate nature of health and healthcare.

To begin to explore the issues associated with translating MI for clinical applications, the National Institutes of Health (NIH) National Center for Advancing Translational Sciences (NCATS) co-hosted a MI in Healthcare workshop with the National Cancer Institute (NCI) and the National Institute of Biomedical Imaging and Bioengineering (NIBIB) on 12 July 2019.

For the purposes of this meeting, MI was defined as the ability of a trained computer system to provide rational, unbiased guidance in such a way that achieves optimal outcomes in a range of environments and circumstances. The meeting held expert panels on the following topics identified as top priorities for healthcare applications, each of which have inherent overlap and complementarity:

- Trustworthiness—the ability to assess the validity and reliability of an MI-derived output across varying inputs and environments. Healthcare professionals need to be able to evaluate the limitations of an MI system and accurately interpret and confidently apply MI-derived information within a clinical setting.
- Explainability—the ability to understand and evaluate the internal mechanism of a machine, algorithm, or computational process in human terms. As these MI systems are being built and applied within a healthcare environment, the development process will need to account for: data quality, quality metrics for the MI system’s functioning and impact, standards for applications in the healthcare environment, and future updates to the data/system.
- Usability—the extent to which an MI system can be used to achieve specified goals with effectiveness, efficiency, and patient satisfaction in multiple healthcare environments. These applications need to be scalable across multiple settings and improve on usual care while preventing additional burden on providers and patients.
- Transparency and fairness—the right to know and understand the aspects of a dataset/input that could influence outputs (clinical decision support) from algorithms. Such factors should be available to the people who use, regulate, and are affected by any type of care decision that employ these algorithms. In healthcare, we need to determine the best ways of identifying and preventing bias in the data—encouraging data transparency and ensuring open access to MI algorithm and system development, all of which have unique challenges in the context of healthcare.

Workshop attendees included scientists, physicians, informaticians, and patients from academic, industry, regulatory, and patient advocacy organizations. The wide range of issues discussed—reflected in this whitepaper—pertained both to the significant potential of MI in improving patient health care and outcomes, and the barriers associated with the development and use of MI in clinical environments. The following sections report on the key issues and challenges identified in each topic area, and potential approaches to address them.

## Results

### Session 1: Trustworthiness of MI in healthcare

For widespread adoption of MI systems and tools in the healthcare space, the professionals employing those tools and the patients for whom they are aiming to benefit must trust their output and reliability. At the highest level, trust is earned when an MI system repeatedly does what it claims to do in delivering valid, reliable answers to important clinical problems. To move the needle, the community needs methods to evaluate output validity, quality, and reliability. However, demonstrating consistent performance can be a particular challenge given the continually evolving nature of MI systems and medical research. Additionally, MI systems—sometimes termed “black boxes” for precisely this reason—are complex and not necessarily fully understandable in how the system produces a particular output. For MI systems to become effective, and consistently accepted and applied within the healthcare setting, the user must be able to trust the system and have confidence that the output provided is correct and appropriate for the situation at hand. Trustworthiness is of particular importance prior to broad uptake of a new technology and needs to be established in:

- The Data—Since many MI systems are built on secondary data sources, “scoring” the data and its fit/quality/relevance for the MI system is integral. An initial step in this direction requires the ability to describe data sets in terms of composition, provenance, representativeness, and completeness.
- The System—Reproducibility^3^ and robustness (e.g., against adversarial examples^4^) of the technical and conceptual aspects of the MI system should be prioritized.
- The Output—An additional system (can be human) must evaluate the trustworthiness of the MI system output^5^.
- The Workflow—The MI component is a small cog in a much larger process known as the overarching MI workflow. Continual surveillance of the MI system is essential even after the system is in use—just like in other industries, there is a need to monitor long-term outcomes and establish structured feedback loops^6^ (i.e., post-market surveillance).

Trust in an MI system requires that we have confidence in all steps from implementation to use (i.e., end-to-end). However, one of the challenges of MI in the healthcare space is that the meaning of trust can change depending on the audience. For example, a machine learning expert expects different validation testing for an MI-supported clinical decision aid than a clinical end-user^7^. Omission of a particular stakeholder or audience during MI application development can often reduce an application’s clinical usefulness, such as when the clinical care environment is not considered during algorithmic development^8^. To promote uptake of MI applications in the clinical care community, providers need: sufficient warning prior to system updates; transparency and understanding of what the MI output means; interpretability through publicly available output benchmarks; and expert review of outputs to ensure reliability of the MI system.

### Session 2: Explainability of MI in healthcare

MI explainability can be broken down into two contrasting types of explanations—interpretability (describing the internals of a system) and completeness (describing the operation of a system). Different levels of interpretability and completeness may be needed depending on the user and their familiarity with MI. Several methodologies for increasing the explainability of MI systems were proposed:

- Explaining the processing of data—examples include generative image transformation^9,10^, proxy systems (an interpretable system that behaves analogously), occlusion visualization, saliency maps, and class activation maps.
- Applying techniques and best practices to increase visibility of inputs that indicate how the system determined the output^11^.
- Illustrating the output as a score assisted by visualization (an explanation interface).
- Creating explanation-producing systems through network dissection, disentangled representations, or explicitly training networks to generate explanations^12^.

Explainability can help motivate development and uptake of therapeutics or diagnostic tests^13^. However, MI applications may not always need explainability, especially if there is a high-level of established trust. For example, in healthcare, many drugs for which the mechanism of action is unknown are trusted due to years of use that illustrate high efficacy and a clean safety profile (e.g., lithium^14^). These historical data do not yet exist with MI applications in healthcare. Therefore, explainability is particularly important at our current point in time in order to encourage acceptance of these MI systems and build trust over time.

### Session 3: Usability of MI in healthcare

There are many types of data sources related to health—e.g., medication lists, demographics, social media, wearables—but, at this time, stakeholders are unable to use them in point-of-care settings as they have yet to be “integrated” appropriately to support the full range of MI applications. Despite major investments in electronic health records (EHRs), the point-of-care setting remains a “walled garden” due to isolated, noninteroperable, and tailored implementation across health care provision sites. However, it has been shown that interoperability can be implemented successfully for large data-dependent systems, such as with the World Wide Web, and there is increasing awareness of the importance of interoperability within digital health^15–17^. New efforts in health IT interoperability are focusing on a handful of well-defined application programming interfaces (APIs). One of these, the Substitutable Medical Applications, Reusable Technologies (SMART) on Fast Healthcare Interoperability Resources (FHIR) API, already standardized through Health Level 7 (HL7) and used extensively by the Centers for Medicare & Medicaid Services (CMS), may soon enable ready access to population data from EHRs without the need for special expertise at each site of care^18,19^. A number of key points were made concerning usability:

- System feedback, such as EHR error prevention responses, is integral^20^, with interfaces being user-centered.
- The current clinical workflow and its constraints need to be understood so MI system guided interventions can be compared to usual care.
- Communication among groups, disciplines, and sectors is essential to ensure development of optimally useful and beneficial systems.

### Session 4: Transparency and fairness of MI in healthcare

Transparency and fairness in healthcare are not new concepts, but new issues arise and are especially acute within MI applications in healthcare. Introduction of bias is a pressing issue that has become a focal point of MI fairness^21,22^. Bias can be introduced into a system in a number of ways, e.g., (1) transfer of MI applications from one population to another with different distributions of features, often affecting protected characteristics with the potential of introducing racial, gender, or social bias; (2) intentional or malicious introduction of bias in order to skew performance; (3) introduction of bias by the data, which is often caused by inadequate or narrowly defined datasets;^23^ (3) the MI system itself can introduce bias based on the structure of the algorithm or the question being posed by the developer; and (4) equipment being used to collect data can introduce bias, through instrument measurement drift, potentially causing significant differences between datasets and outputs. These, as well as other forms of bias not listed here, must be assessed and addressed in order to prevent their effects and amplification.

Several solutions to this issue were suggested, including (1) tracking and education around biased language, (2) development of approaches to identify bias in results, (3) utilization of representative datasets, and (4) testing and validation of algorithms across multiple health systems and/or datasets. Transparency and scrutiny of data alone is unfeasible and ignores the wider behavioral and systemic sources of automated and amplified bias. Ultimately, the field will need to work toward transparency of processes and frameworks as we uncover new sources of bias in automated systems and MI.

Thinking of MI applications in terms of equality and equity of healthcare, treatment, and outcomes is crucial. Currently, the quality of treatment among patients is not equal^24^, which can exacerbate disparities when these MI systems are not uniformly implemented. At the same time, there is a lack of equity among health systems—with patients in one area experiencing difficulty in accessing the same resources and tools that are easily available in another area. Lastly, the goal of achieving commensurate outcomes may have significant effects on the generalizability and appropriate application of MI systems in healthcare—e.g., two patients may be prescribed the same drug, but respond differently, requiring that patients receive treatments tailored to their individual needs.

## Discussion

### Gaps, barriers, and methodologies for implementation of MI in healthcare

#### The data

In fields in which the use of MI is more mature, high-quality training and test data are understood to be fundamental; that is, MI outputs can be trusted only if the input data are trusted. Many MI systems in healthcare are being built on secondary data (such as real-world data), which has not been collected specifically for the MI system of interest and therefore can introduce unanticipated issues. But even MI systems built on primary data sources, such as remote monitoring, require a high level of scrutiny. Some suggested methodologies to address these issues in the data include:

- “Scoring” of the data—data can be inaccurate and/or incomplete; to aid in overcoming this and enable the creation of targeted data standards for MI applications, data could be scored for quality and applicability to the MI system.
- Comparison of data to other relevant benchmark data sets and studies, as well as the results from current usual care^25^.
- Automated extraction of unstructured data using an API to assist healthcare systems in the generation of structured (and therefore usable) data.

Data potentially relevant to healthcare MI are being generated at a prodigious rate, increasing the need for updates to MI datasets and algorithms, and vigilant testing for bias introduced by these updates. Particular issues are presented by the use of published data, which can be unvalidated or incomplete due to selective reporting^26,27^. Options to mitigate introduction of bias include:

- Development of new linguistic markers of bias with the ultimate goal of developing quantitative methods (e.g., natural language processing) that overlay the EHR.
- Use of representative datasets to help avoid bias in results.
- Development of standard protocols for research work and clinical applications; and replication of outputs in multiple situations^28^ to reduce bias and prevent its perpetuation.

#### The Infrastructure

EHR systems have now been implemented in most advanced healthcare environments, but each instance tends to be unique even if the same overall EHR (e.g., Epic, Cerner) is used, further limiting interoperability. Pulling data (whether it be reference population or otherwise) out of EHRs into analytic platforms can therefore be a problem for small groups, and is often a major challenge for large hospital/health systems. Additionally, many other data-related issues exist for the application of MI in healthcare settings, such as: systemic, missing data; complex integration of secondary data sources; and limited data structures, metadata, and visualization features. Possible solutions might include:

- An API layer that sits on top of the EHR system, running across individual instances of the EHR to connect health systems data—e.g., the SMART on FHIR API layer connects health systems data to a software application (app) layer^29^.
- An emerging API, Clinical Decision Support (CDS) Hooks, enables the launch of MI applications at the appropriate point in the physician’s workflow—it allows the EHR to call a third party decision service or launch a SMART on FHIR app.
- A new standard for accessing data on whole populations rather than one patient at a time—the SMART/HL7 FHIR bulk data^30^ export enables population data exchange, via an API, between provider organizations and third parties.

#### The MI workflow

Reliable data and algorithms are absolutely necessary, but are not sufficient, for implementation of MI in clinical settings. Application of MI requires comprehension and assessment of its implications to clinicians, patients, and other members of the care team—all while testing and adapting in real-time based on real patient data and situations. Increased collaboration among scientific/medical/technical disciplines and sectors is needed around development of the MI workflow and training data, with outputs tailored to the user, to ensure that these systems can be trusted and implemented. In addition, MI systems must also undergo updates when new data are available and/or if improvements need to be made to the algorithm, requiring continual MI workflow monitoring. After integration, these MI systems will need to be monitored to ensure they are running properly—no matter the modality of MI system used. Finally, different levels of MI workflow explanation will be required depending on the target user and audience.

#### The MI system

It will be critical to share with stakeholders how MI systems operate in order to allow users to evaluate and trust the systems’ output. This can be achieved several ways:

- Expose the training data and labels used to train the system (with privacy in mind), allowing others to reproduce the output.
- Apply techniques like attention markers, confidence scores, and/or output visualizations to increase understanding of the outputs and how the input data affect those outputs.
- Foster an understanding of the tradeoffs for false positives and false negatives for any prediction/inference.
- Create a systematic review system to conduct ongoing review of the current literature, which will provide clinicians with trusted medical evidence for comparison with MI system outputs.
- Reproduce the result consistently on new samples^31^.

While there is a desire to have MI systems that work across healthcare provision sites, it is unlikely that the same system will meet the needs of multiple locations. For example, it is highly unlikely that a mortality prediction system at a hospital in California will work in the same way when implemented half-way around the world, particularly due to site-specific variations^32,33^. For implementation of these MI systems, robustness of the system must be tested, meaning that a user must understand when to rely on or reject a system output, what populations the system might work or fail on, and limitations of the data on which the system is trained. Robustness can be evaluated: (1) through the use of adversarial examples; (2) against publicly available benchmarks; and (3) against the context of the output—there is a need to explore causality in these MI systems and the context within which outputs are determined.

### Overarching issues and conclusions

There were several overarching issues associated with MI applications in healthcare that surfaced:

- Crisper, clearer, and more streamlined definitions for common MI terms are needed. With the same terminology used for development across the entire space, practitioners and users in different fields will be able to communicate more meaningfully.
- Developers should focus on asking questions that are relevant to the user and can be acted upon. Explainable outputs are not necessarily actionable or informative outputs.
- The comparative effectiveness of MI systems should be evaluated against usual care and other health outcomes.
- There is a need for interdisciplinary collaboration—focusing not on the science alone, but also (and especially) on how the system will be implemented and who will use it (implementation science), and pragmatic trial design to work within the complexity of the healthcare domain. A convergence between expertise and sectors will be required.
- Clinicians and patients should be involved in the development of such MI systems if they are envisioned as a user. Patient and public engagement is integral to achieve trustworthiness of MI systems in healthcare.
- Appropriate and ethically sound data security and privacy measures should be developed and consistently implemented with fairness and transparency in mind.
- Creation of approaches for critical appraisal of MI systems is needed before they are deployed in healthcare settings. This will require safe spaces to cover end-to-end MI system deployment, with continual surveillance after implementation.
- Long-term MI system outcomes must be monitored and used to establish structured feedback loops for continual improvement.

MI approaches have the potential to improve health through the facilitation of more efficient and effective provision of care. But given the many scientific, operational, cultural, and ethical issues that must be resolved for that potential to be realized, it is imperative for us all—the many stakeholders in the healthcare ecosystem—to work together to translate MI tools for clinical and healthcare applications.

## Data Availability

Data sharing is not applicable to this article as no datasets were generated or analyzed during the current study. No code or mathematical algorithm was developed for this manuscript.
